# Novel Computational Methods and Tools in Biomedicine and Biopharmacy

**DOI:** 10.1155/2014/127515

**Published:** 2014-06-05

**Authors:** Yudong Cai, Tao Huang, Lei Chen, Shan Gao, Ning Zhang

**Affiliations:** ^1^Institute of Systems Biology, Shanghai University, Shanghai 200444, China; ^2^Department of Genetics and Genomics Sciences, Mount Sinai School of Medicine, New York, NY 10029, USA; ^3^College of Information Engineering, Shanghai Maritime University, Shanghai 201306, China; ^4^Boyce Thompson Institute for Plant Research, Cornell University, Ithaca, NY 14853, USA; ^5^Department of Biomedical Engineering, Tianjin Key Laboratory of BME Measurement, Tianjin University, Tianjin 300072, China

With the rapid development of new biomedical and biopharmacy data collection technologies, it is desired to develop corresponding methods and tools for analyzing these big data with various structures. Such efforts can help deriving important information and knowledge from these data to promote the development of biomedicine and drug design.

In this special issue, eight interesting studies were included. Novel methods were proposed for analyzing unconventional data, such as biomedical annotation and annotator data, PET/CT Images. And useful tools were developed for interdisciplinary research, such as facial visualization system.

Q.-D. Fan et al. analyzed the cell apoptosis network at posttranslational level with time delay differential equations. Their results suggest that posttranslational modifications of p53 have different dynamics and functions. The article provides a dynamical insight of p53-induced cell repair and cell apoptosis.

A. Wu proposed a weighted and concept-extended resource description framework (RDF) model to rank the annotations by evaluating their correctness according to user's vote and the semantic relevancy between the annotator and the annotated entity. This approach is applicable and efficient even when data set is large.

W. Kong et al. reconstructed the signaling pathways of Alzheimer's disease by combining protein-protein interaction (PPI) data with gene expression data. They found that the genes on the reconstructed pathways play crucial roles in inflammatory response and APP (amyloid precursor protein).

W. Hongwei et al. described a new data visualization system by plotting the human face to observe the comprehensive effects of multivariate data. The graphics device interface (GDI+) in the Visual Studio.NET development platform was used to generate facial image according to* Z* values from sets of normal data.

Y. Guo et al. proposed a robust method for automatic lung tumor segmentation on PET/CT image. This method is based on fuzzy Markov random filed model and can achieve effective lung tumor segmentation even when tumors locate near other organs with similar intensities in PET and CT images.

H. Zhao et al. employed the hexaMplot to illustrate the continuous variation of the gene expressions of the embryonic cells treated with the different doses of tachyplesin I (TP I). The technology of hexaMplot was proved to be an intuitive and effective tool to illustrate the genetic interrelations in microarray analysis.

Y. Cui et al. proposed a novel case allocation system, MACT, using minimization method. This system employs a simplified database and has a unified interface that manages trials, participants, and allocations. Applications show that MACT is stable, manageable, and easy-to-use. Its outstanding features are attracting more random clinical trials.

X. Qiu et al. proposed a hybrid method Quad-PRE to predict protein quaternary structure attributes using the properties of amino acid, predicted secondary structure, predicted relative solvent accessibility, position-specific scoring matrix profiles, and motifs. The overall accuracy of Quad-PRE is 81.7%. Quad-PRE can classify protein quaternary structure attributes effectively.

As more biomedical and biopharmacy data will be generated in the future and the data structure will be even more complex, the methods and tools in this special issue may become important and inspire other researchers.

*Yudong Cai* *Yudong Cai* *Tao Huang* *Tao Huang* *Lei Chen* *Lei Chen* *Shan Gao* *Shan Gao* *Ning Zhang* *Ning Zhang*

